# RGB-D Object Recognition Using Multi-Modal Deep Neural Network and DS Evidence Theory

**DOI:** 10.3390/s19030529

**Published:** 2019-01-27

**Authors:** Hui Zeng, Bin Yang, Xiuqing Wang, Jiwei Liu, Dongmei Fu

**Affiliations:** 1 School of Automation and Electrical Engineering, University of Science and Technology Beijing, Beijing 100083, China; g20178627@xs.ustb.edu.cn (B.Y.); liujiwei@ustb.edu.cn (J.L.); fdm_ustb@ustb.edu.cn (D.F.); 2 Beijing Engineering Research Center of Industrial Spectrum Imaging, Beijing 100083, China; 3 Vocational & Technical Institute, Hebei Normal University, Shijiazhuang 050024, China; xqwang@hebtu.edu.cn

**Keywords:** RGB-D object recognition, deep neural network, multi-modal learning, DS evidence theory

## Abstract

With the development of low-cost RGB-D (Red Green Blue-Depth) sensors, RGB-D object recognition has attracted more and more researchers’ attention in recent years. The deep learning technique has become popular in the field of image analysis and has achieved competitive results. To make full use of the effective identification information in the RGB and depth images, we propose a multi-modal deep neural network and a DS (Dempster Shafer) evidence theory based RGB-D object recognition method. First, the RGB and depth images are preprocessed and two convolutional neural networks are trained, respectively. Next, we perform multi-modal feature learning using the proposed quadruplet samples based objective function to fine-tune the network parameters. Then, two probability classification results are obtained using two sigmoid SVMs (Support Vector Machines) with the learned RGB and depth features. Finally, the DS evidence theory based decision fusion method is used for integrating the two classification results. Compared with other RGB-D object recognition methods, our proposed method adopts two fusion strategies: Multi-modal feature learning and DS decision fusion. Both the discriminative information of each modality and the correlation information between the two modalities are exploited. Extensive experimental results have validated the effectiveness of the proposed method.

## 1. Introduction

Object recognition is one of the fundamental problems in the fields of computer vision and robotics. Until now, many methods have been proposed for object recognition, but most of them are based on the RGB (Red Green Blue) image. However, the RGB image can only reflect the color, illumination, and texture information of the scene, and the depth information of the scene is lost during the optical projection process from the 3D (Three Dimensional) space to the 2D (Two Dimensional) space. Therefore, RGB image based object recognition methods are susceptible to external factors, such as illumination and a complex background, which significantly impede the usage of the RGB image based object recognition methods in practical applications [1,2,3,4,5].

In recent years, with the development of low-cost RGB-D (Red Green Blue-Depth) sensors, such as Microsoft Kinect and Intel RealSense, the RGB-D image has been widely used in scene analysis and understanding, video surveillance, intelligence robot, and medical diagnosis [6,7]. The RGB-D sensor can capture the color image and the depth image at the same time. The RGB image contains color and appearance information, and the depth image contains the distance information between the RGB-D sensor and the object. Compared with RGB image, the RGB-D image can provide additional information about the 3D geometry structure of the object, which has more effective information for object recognition. Furthermore, the depth image is robust to variations in color and illumination. It has been proven that the RGB-D image based object recognition method can achieve better performance than the RGB image based object recognition method. So, the research of the RGB-D image based multi-modal object recognition method has attracted more and more attention in the last few years [8,9,10].

According to the types of the features, existing RGB-D image based object recognition methods can be divided into two categories: Hand-crafted feature based methods and learned feature based methods. For the first category, the hand-crafted features, such as scale-invariant feature transform (SIFT) [11], speeded up robust features (SURF) [12], and spin images [13,14], are extracted to describe the RGB and depth images, respectively, and then they are fed into the classifiers, such as SVMs (Support Vector Machines), for classification. The performance of this kind of method is influenced by the selected hand-crafted features. The hand-crafted features often need to be manually tuned for different conditions, and they cannot capture all the useful discriminative information of different classes of objects. For the second category, the features are learned from the RGB and depth images, and then the classifiers are used for classification. This kind of method performs better, but it still does not make full use of the effective information contained in the RGB-D images. Most existing methods usually learn separately from the RGB and depth images, and the two kinds of features are simply combined for recognition [15,16]. So how to make full use of the relationship of the RGB features and the depth features is still a key problem to be solved in the field of RGB-D object recognition.

The DS (Dempster Shafer) evidence theory is a useful uncertain reasoning method for multi-sensor information fusion [17,18]. It can be regarded as a generalization of the Bayes theory of subjective probability, which can grasp the uncertainty of the problem and performs better than the traditional probability theory. The DS evidence theory has been successfully used in pattern recognition, expert system, fault diagnosis, and information fusion [19,20]. In this paper, two SVM classifiers are used for RGB modality and depth modality, and we use the DS evidence theory to fuse the decisions of two classifiers. Compared with the weighted fusion method, the DS evidence theory based decision fusion method considers the effects of different decisions for different classes by using the mass function, which can give more reasonable recognition results.

In this paper, we focus on a multi-modal deep neural network and DS evidence theory based RGB-D object recognition methods. First, the RGB and depth images are preprocessed and three channel images of them are obtained as the inputs of each convolutional neural network (CNN). Second, the RGB CNN and the depth CNN are trained using the stochastic gradient descent (SGD) method with back-propagation. Then, the multi-modal feature learning network is trained to fine-tune the network parameters, where the objective function includes both the discriminative terms and the correlation term. Finally, we construct two support vector machine (SVM) classifiers for each modality, and the DS evidence theory is used to fuse the two decision results. To summarize, the contributions of this paper include:

- The CNN based multi-modal deep neural network is built for learning RGB features and depth features. The training of the proposed multi-modal network has two stages. First, the RGB CNN and the depth CNN are trained, respectively. Then, the multi-modal feature learning network is trained to fine-tune the network parameters;
- we propose a quadruplet samples based objective function for each modality, which can learn the discriminative feature more effectively. Furthermore, we propose a comprehensive multi-modal objective function, which includes two discriminative terms and one correlation term; and
- for each modality, an effective weighted trust degree is designed according to the probability outputs of the two SVMs and the learned features. Then, the total trust degree can be computed using the Dempster rule of combination for object recognition.

The rest of this paper is organized as follows. Section 2 provides a brief overview of the related work. Section 3 introduces the proposed RGB-D based object recognition method in detail, including RGB-D image preprocessing, the architecture, and the learning method of the proposed multi-modal feature learning method and the DS evidence theory based RGB-D object recognition method. Section 4 reports the experimental results and the detailed comparable analysis. Finally, conclusions are provided in Section 5.

## 2. Related Work

Remarkable efforts have been investigated to explore RGB-D image based object recognition in recent years. Earlier works mainly focus on hand-crafted feature based methods. For example, Lai et al. used spin images for depth images and SIFT descriptors for RGB images [21]. At first, the spin images of the sampled 3D points were computed, and then the efficient match kernel (EMK) features were obtained to describe the entire shape. Then, the SIFT descriptors were extracted and their corresponding EMK features were computed. Additionally, the texton histograms were also extracted to capture texture information. Finally, three state-of-the-art classifiers, including the linear support vector machine (LinSVM), Gaussian kernel support vector machine (kSVM), and random forest (RF), were used for recognition. Bo et al. proposed five depth kernel descriptors (gradient kernel, spin kernel, size kernel, kernel principal component analysis (PCA), and local binary pattern kernel) to capture different recognition cues, including size, shape, and edges, which can significantly improve the performance of RGB-D object recognition [22]. Yu et al. proposed a kind of continuous local descriptor called local flux feature (LFF), which can be used for both an RGB image and depth image [23]. Then, the structure preserving projection (SPP) was used to fuse RGB information and depth information, and a novel binary local representation was obtained for RGB-D data. Logoglu et al. proposed two spatially enhanced local 3D descriptors: Histograms of spatial concentric surflet-pairs (SPAIR) and colored SPAIR (CoSPAIR) [24]. The CoSPAIR descriptor contains both shape information and color information, and it performs well in RGB-D object recognition. In summary, the hand-crafted features were designed according to part characteristics of the objects, and they cannot satisfy the needs of the RGB-D object recognition of a large-scale dataset.

Compared with the hand-crafted feature based methods, the learned feature based methods have achieved better performance and have attracted more and more researchers’ attentions. For example, Bo et al. proposed a feature learning method for RGB-D based object recognition by making hierarchical matching pursuit (HMP) for color and depth images [25]. HMP uses sparse coding to learn hierarchical feature representations from raw RGB-D data in an unsupervised way. Blum et al. proposed a new learned local feature descriptor for RGB-D images, called the convolutional k-means descriptor [26]. It automatically learns feature responses in the neighborhood of detected interest points and is able to combine color information and depth information into one concise representation. Asif et al. proposed a bag-of-words (BOW) based feature learning method for RGB-D object recognition [27]. The randomized clustering trees were used to learn visual vocabularies, and the standard spatial pooling strategies were used for feature representation. Huang et al. proposed a discriminative cross-domain dictionary learning based RGB-D object recognition framework, which learns a domain adaptive dictionary pair and classifier parameters in the data representation level and classification level, respectively [28]. Li et al. proposed an effective multi-modal local receptive field extreme learning machine (MM-ELM-LRF) structure for RGB-D object recognition [29]. The extreme learning machine (ELM) was used as a supervised feature classifier for the final decision, and the proposed MM-ELM-LRF method maintains ELM’s advantages of training efficiency. In general, most of the above learned feature based methods learn features from the color images and the depth images separately. Thus, the correlation information between the two modalities has not been fully exploited.

Recently, deep learning has become extremely popular and has been successfully applied in RGB-D object recognition. Socher et al. proposed a model based on a combination of convolutional and recursive neural networks (CNN and RNN) for learning features and classifying RGB-D images [30]. The CNN layer learns low level features, and the RNN layer composes higher order features. Wang et al. proposed a general CNN based multi-modal learning method for RGB-D object recognition, which can simultaneously learn transformation matrices for two modalities with a large margin criterion and a maximal cross-modality correlation criterion [31]. Rahman et al. proposed a three-stream multi-modal CNNs based deep network architecture for RGB-D object recognition [32]. The three streams include surface normal, color jet, and RGB channel. Tang et al. proposed a canonical correlation analysis (CCA) based multi-view convolutional neural networks for RGB-D object recognition, which can effectively identify the associations between different perspectives of the same shaped model [33]. Zia et al. proposed a hybrid 2D/3D convolutional neural network for RGB-D object recognition, which can be initialized with pretrained 2D CNN and can be trained over a relatively small RGB-D dataset [34]. Bai et al. proposed a subset based deep learning method for RGB-D object recognition [9]. At first, the raw dataset was divided into some subsets according to their shapes and colors. Then, two sparse auto-encoders were trained for each subset, and the recursive neural network was used to learn robust hierarchical feature representations. Finally, the learned features were sent to a softmax classifier for object recognition. Although the above methods have achieved good performance, learning effective discriminative information from the RGB-D images is also a problem worthy of further research.

Furthermore, there are many good performing methods in the RGB-D tracking literature, which are introduced to fuse color and depth channels. Song et al. proposed an RGB-D histogram of oriented gradients (HOG) feature based method for RGB-D tracking [35]. The RGB-D HOG features can describe local textures as well as 3D shapes. Furthermore, they also proposed the second tracking method [35], which is based on the 3D point cloud. They designed the point cloud feature to capture the color and shape of cells of 3D points. Both methods used sliding window detection with linear SVM, and the 2D optical flow method and 3D iterative closest point (ICP) method were adopted for point tracking. Meshgi et al. proposed an occlusion aware particle filter tracker based on RGB-D images [36], which employs a probabilistic model with a latent variable representing an occlusion flag. The probabilistic framework accommodates the adaptive fusion of the features extracted from RGB and depth images. Camplani et al. proposed a real-time RGB-D tracking with depth scaling kernelised correlation filters and occlusion handling [37]. They fused color and depth cues as the tracker’s features by evaluating a variety of feature combination strategies.

## 3. Proposed Method

**Figure 1:**
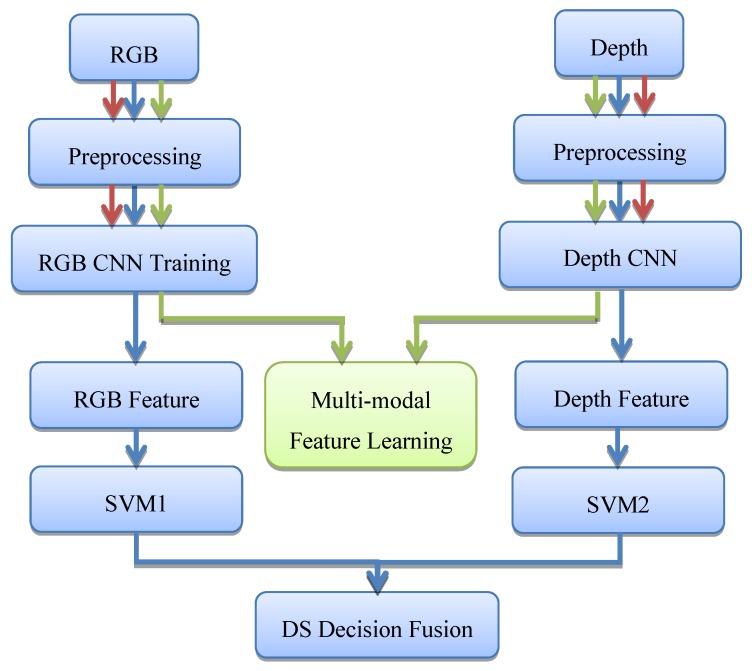
The flowchart of the proposed RGB-D object recognition method.

**Figure 2:**
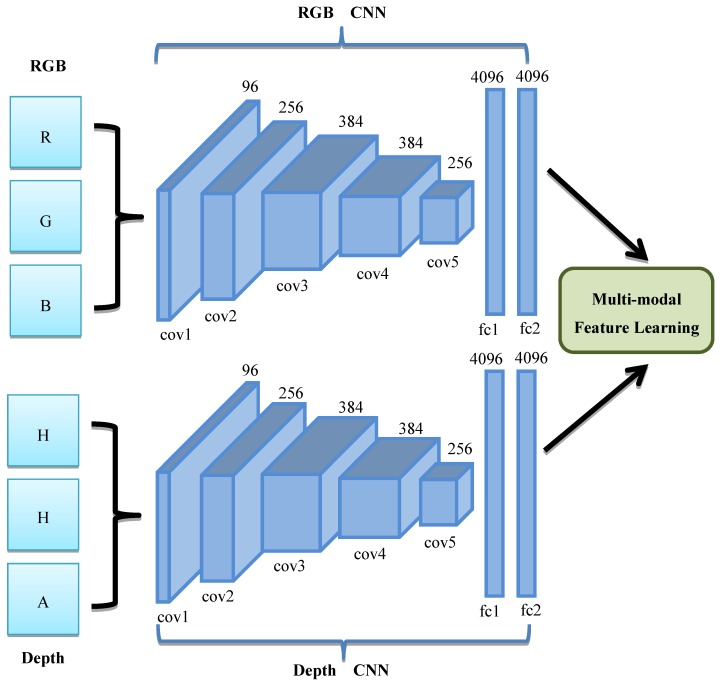
The architecture of the proposed multi-modal network.

As shown in Figure 1, our proposed RGB-D object recognition method has three pipelines. The red pipeline and the green pipeline are used for training the CNNs, and the green pipeline is used for testing. In the training stage, the RGB image and the depth image are first preprocessed to reduce noises, and they are rescaled to the normalized size. Next, we compute three channels of the depth image using the HHA encoding method [38,39,40,41,42], where the HHA code refers to the horizontal disparity, height above ground, and angle with gravity. The red pipeline is used to train each CNN, respectively, and the trained network parameters of the two CNNs are used as the initial parameters of the following multi-modal feature learning. Then, we use the green pipeline to perform multi-modal feature learning using the two CNNs. Through multi-modal learning, the parameters of the two CNNs can be optimized according to both the correlation information between the two modalities and the discriminative information in each modality. In the testing stage, we use the blue pipeline for RGB-D object recognition. The optimized parameters of the two CNNs are used for computing the RGB features and the depth features. After the learned RGB and depth features of the testing sample have been computed, two SVM classifiers are used for each modality. Finally, the DS evidence theory is used to fuse the two recognition results. Figure 2 gives the architectures of the two CNNs (RGB CNN and depth CNN), which are the same as the AlexNet [43]. Our experimental results have shown that both the proposed multi-modal feature learning strategy and the DS fusion strategy can improve the recognition efficiency.

### 3.1. RGB-D Image Preprocessing

**Figure 3:**
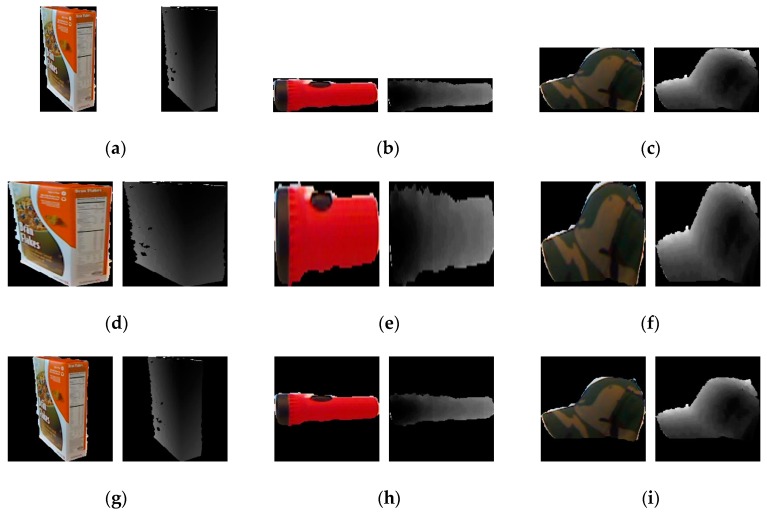
The results of image scaling. (**a**) The RGB and depth images from the “ceteal_box” class; (**b**) the RGB and depth images from the “flashlight” class; (**c**) the RGB and depth images from the “cap” class; (**d**) the resized images of (**a**); (**e**) the resized images of (**b**); (**f**) the resized images of (**c**); (**g**) the scaled images of (**a**); (**h**) the scaled images of (**b**); (**i**) the scaled images of (**c**).

To meet the requirements of the two CNNs, which use the basic architecture of AlexNet, the input RGB and depth images are first scaled to 227 × 227. The simplest way is to resize the images to the required image size directly. However, as shown in Figure 3b–f, the direct method may deform the object’s original ratio and geometric structure, which will influence the recognition performance. So, we used the scaling processing method proposed in [33]. At first, we resized the origin image so that the length of its long side becomes 227 pixels. Then, we expanded the resized image along the short side to obtain a square image. The two sides of image expansion should be equal and the resized image should be located in the middle of the expansion scaled image. The expansion of the images is done by adding black pixels. Figure 3g–i shows the scaled images. Form Figure 3, we can see that compared with the resized images, the scaled images can effectively preserve the shape information of the objects.

For the scaled RGB image, we can obtain its R channel image, G channel image, and B channel image as three input images of the RGB CNN. For the scaled depth image, we first fill out its holes and reduce noise using the median filters. Then, the HHA encoding method is used to obtain three input images of the depth CNN. The HHA representation can encode the properties of the geocentric pose that emphasize complementary discontinued in the image, and has been successfully used in several RGB-D image based works [38,39,40,41,42].

### 3.2. Feature Learning Method of the Proposed Multi-Modal Network

#### 3.2.1. The Architecture of the Proposed Multi-Modal Network

The proposed multi-modal network is designed to extract the features of the RGB and depth images. Figure 2 illustrates the architecture of the proposed multi-modal network, which consists of two branches. Each branch is a CNN with the same architecture as the AlexNet [43]. The inputs of the first branch are the three channels of the RGB images, and the inputs of the second branch are the HHA encoding results of the depth images. The AlexNet consists of five convolutional layers and three fully-connected layers with a final 1000-way softmax. It has about 60 million parameters and 650,000 neurons. In this paper, we only used five convolutional layers and the first two fully-connected layers. The first convolutional layer, the second convolutional layer, and the fifth convolutional layer are followed by max-pooling layers. The activation function of all convolutional layers and fully-connected layers is the rectified linear unit (ReLU). The last fully-connected layer is deleted and the second fully-connected layer is used for feature extraction. The training of the proposed network has two stages. In the first stage, the RGB feature and the depth feature are learned separately using their corresponding CNNs. In the second stage, the multi-modal network is fine-tuned using the RGB and depth images. Both the discriminative information of each modality and the correlation information between two modalities are considered in the optimization process.

#### 3.2.2. RGB Feature Learning and Depth Feature Learning

In this paper, we first learn the RGB features and the depth features, respectively. Here, we elaborate on the objective function for learning the RGB features, and likewise for learning the depth features. Inspired by the deep quadruplet network proposed for learning a local feature descriptor, we designed a novel quadruplet samples based objective function. Compared with the triplet objective function, the quadruplet objective function is less prone to over-fitting and has a better training efficiency [44,45]. The difference between the existing deep quadruplet network and our work is that the deep quadruplet network has four branches. We have not adopted the four-branch network structure and only used the concept of quadruplet in the objective function. The aim of our proposed quadruplet objective function is to minimize intra-class distances and maximize inter-class distances.

Assume $\left(x_{i},x_{j},x_{k},x_{l}\right)$ is a sample quadruplet, which is obtained using the sampling method proposed in Ref. [39]. Among them, the sample, $x_{i}$ and $x_{j}$, are from the same class, and they are called a positive sample pair. The sample, $x_{k}$ and $x_{l}$, are from different classes, and they are called a negative sample pair. The positive set, P, contains a number of positive sample pairs, and the negative set, N, contains a number of negative sample pairs. For the input sample, x, let $f_{1}(x)$ be its output of the second fully-connected layer of the RGB CNN, which is the learned RGB feature. Then, the quadruplet objective function can be defined as:

(1) $$\underset{\rho_{1}}{\min}F_{1}=\sum_{(i,j)\in P}h\left({\left\|f_{1}(x_{i})-f_{1}(x_{j})\right\|}_{2}^{2}-T_{1}\right)+\rho_{1}\sum_{(k,l)\in N}h\left(T_{1}+\tau_{1}-{\left\|f_{1}(x_{k})-f_{1}(x_{l})\right\|}_{2}^{2}\right)$$

where h is a hinge loss function, h(x)=max(0,x), and $\rho_{1}$ is the weight. From Equation (1), we can conclude that the proposed quadruplet objective function can make the distance between the positive sample pair, $\left(x_{i},x_{j}\right)$, smaller than a given threshold, $T_{1}$, and it also can make the distance between the negative sample pair, $\left(x_{k},x_{l}\right)$, larger than a given threshold, $T_{1}+\tau_{1}$. In summary, our proposed objective function encourages that the distances between the same-class samples to be smaller by at least the margin, $\tau_{1}$, than the distances between different-class samples.

In this paper, the RGB CNN is initialized using transfer learning. The initial parameters are obtained from pretrained AlexNet on the ImageNet large scale dataset. Then, we fine-tuned the RGB CNN, which is trained by the SGD method with back-propagation, and the derivatives of the loss function, $F_{1}$, with respect to $\rho_{1}$ $f_{1}(x_{i})$, $f_{1}(x_{j})$, $f_{1}(x_{k})$, and $f_{1}(x_{l})$ can be derived as:

(2) $$\frac{\partial F_{1}}{\partial \rho_{1}}=\sum_{(k,l)\in N}h\left(T_{1}+\tau_{1}-{\left\|f_{1}(x_{k})-f_{1}(x_{l})\right\|}_{2}^{2}\right)$$

(3) $$\frac{\partial F_{1}}{\partial f_{1}(x_{i})}=2\sum_{j}\left[f_{1}(x_{i})-f_{1}(x_{j})\right]\cdot h^{\prime }\left({\left\|f_{1}(x_{i})-f_{1}(x_{j})\right\|}_{2}^{2}-T_{1}\right)$$

(4) $$\frac{\partial F_{1}}{\partial f_{1}(x_{j})}=-2\sum_{i}\left[f_{1}(x_{i})-f_{1}(x_{j})\right]\cdot h^{\prime }\left({\left\|f_{1}(x_{i})-f_{1}(x_{j})\right\|}_{2}^{2}-T_{1}\right)$$

(5) $$\frac{\partial F_{1}}{\partial f_{1}(x_{k})}=-2\rho_{1}\sum_{l}\left[f_{1}(x_{k})-f_{1}(x_{l})\right]\cdot h^{\prime }\left(T_{1}+\tau_{1}-{\left\|f_{1}(x_{k})-f_{1}(x_{l})\right\|}_{2}^{2}\right)$$

(6) $$\frac{\partial F_{1}}{\partial f_{1}(x_{l})}=2\rho_{1}\sum_{k}\left[f_{1}(x_{k})-f_{1}(x_{l})\right]\cdot h^{\prime }\left(T_{1}+\tau_{1}-{\left\|f_{1}(x_{k})-f_{1}(x_{l})\right\|}_{2}^{2}\right)$$

In the optimization process, the weight, $\rho_{1}$, can be updated using Equation (2) and the back-propagation is conducted using Equations (3)–(6).

For the depth CNN, it is initialized using the same initialization method as the RGB CNN. Let $f_{2}(x)$ be the output of the second fully-connected layer of the sample, x, which is the learned depth feature. Similar to the definition of the objective function of the RGB CNN, the objective function of the depth CNN can be defined as:

(7) $$\underset{\rho_{2}}{\min}F_{2}=\sum_{(i,j)\in P}h\left({\left\|f_{2}(x_{i})-f_{2}(x_{j})\right\|}_{2}^{2}-T_{2}\right)+\rho_{2}\sum_{(k,l)\in N}h\left(T_{2}+\tau_{2}-{\left\|f_{2}(x_{k})-f_{2}(x_{l})\right\|}_{2}^{2}\right)$$

where $\rho_{2}$ is the weight, $T_{2}$ and $\tau_{2}$ are the given threshold. Then, the derivatives of the objective function, $F_{2}$, can be derived, and the expressions of $\frac{\partial F_{2}}{\partial \rho_{2}},$ $\frac{\partial F_{2}}{\partial f_{2}(x_{i})},$ $\frac{\partial F_{2}}{\partial f_{2}(x_{j})},$ $\frac{\partial F_{2}}{\partial f_{2}(x_{k})},$ $\frac{\partial F_{2}}{\partial f_{2}(x_{l})}$ are similar to Equations (2)–(6). Finally, the depth CNN can be optimized using the SGD method with back propagation.

#### 3.2.3. Multi-Modal Feature Learning

As the RGB image and depth image of the same object have some implicit relations, we exploited the correlation information of the two modalities to extract more effective features. Inspired by the processing method proposed in Ref. [31], we used the distances between different modalities to construct the correlation term of the objective function. The aim of our proposed correlation term is to maximize the inter-modality relationship of intra-class samples and minimize the inter-modality relationship of inter-class samples. That is to say, we should minimize the distances between the RGB feature and the depth feature of the same class and maximize the distances between the RGB feature and the depth feature of different classes. So, the correlation term can be defined as:

(8) $$F_{c}=\sum_{(i,j)\in P}\left[{\left\|f_{1}(x_{i})-f_{2}(x_{j})\right\|}_{2}^{2}+{\left\|f_{2}(x_{i})-f_{1}(x_{j})\right\|}_{2}^{2}\right]-\mu \sum_{(k,l)\in N}\left[{\left\|f_{1}(x_{k})-f_{2}(x_{l})\right\|}_{2}^{2}+{\left\|f_{2}(x_{k})-f_{1}(x_{l})\right\|}_{2}^{2}\right]$$

where μ is the weight to adjust the influences of the inter-class samples and the intra-class samples. The derivatives of the correlation term can be derived as follows:

(9) $$\frac{\partial F_{c}}{\partial \mu }=-\sum_{(k,l)\in N}\left[{\left\|f_{1}(x_{k})-f_{2}(x_{l})\right\|}_{2}^{2}+{\left\|f_{2}(x_{k})-f_{1}(x_{l})\right\|}_{2}^{2}\right]$$

(10) $$\frac{\partial F_{c}}{\partial f_{1}(x_{i})}=2\sum_{j}\left[f_{1}(x_{i})-f_{2}(x_{j})\right]$$

(11) $$\frac{\partial F_{c}}{\partial f_{2}(x_{i})}=2\sum_{j}\left[f_{2}(x_{i})-f_{1}(x_{j})\right]$$

Similar to Equations (10) and (11), the expressions of $\frac{\partial F_{c}}{\partial f_{1}(x_{j})},$ $\frac{\partial F_{c}}{\partial f_{2}(x_{j})},$ $\frac{\partial F_{c}}{\partial f_{1}(x_{k})},$ $\frac{\partial F_{c}}{\partial f_{2}(x_{k})},$ $\frac{\partial F_{c}}{\partial f_{1}(x_{l})}$, and $\frac{\partial F_{c}}{\partial f_{2}(x_{l})}$ also can be derived.

Finally, we used the discriminative terms of each modality and the correlation term between the two modalities to construct the multi-modal objective function. The objective function, $F_{1}$, can be used as the discriminative term of the RGB modality, and the objective function, $F_{2}$, can be used as the discriminative term of the depth modality. The multi-modal objective function can be expressed as:

(12) $$\begin{array}{l} \underset{\left\{\rho_{1},\rho_{2},\mu ,\lambda_{1},\lambda_{2}\right\}}{\min}F=\lambda_{{}_{1}}^{p}F_{1}+\lambda_{{}_{2}}^{p}F_{2}+\beta F_{c}(\mu )\\ \mathrm{subject}\;\mathrm{to}\;\lambda_{1}+\lambda_{2}=1,\lambda_{1}\geq 0,\lambda_{2}\geq 0,\beta >0,p>1 \end{array}$$

where $\lambda_{1}$ and $\lambda_{2}$ are the weights between the RGB modality and the depth modality, and β is the weight between the discriminative terms and the correlation term. The parameter, p, is the relaxation factor, which can make the discriminative term of both modalities effective [31]. Assume $F_{1}$ and $F_{2}$ are kept constant, if $F_{1}$ is more than $F_{2}$, then the solutions of $\lambda_{1}$ and $\lambda_{2}$ are: $\lambda_{1}=0$ and $\lambda_{2}=1$, which means that only the depth modality is effective; if $F_{1}$ is less than $F_{2}$, then the solutions of $\lambda_{1}$ and $\lambda_{2}$ are: $\lambda_{1}=1$ and $\lambda_{2}=0$, which means that only the RGB modality is effective. For the above two conditions, only one modality is effective and the correlation information of the two modalities cannot be exploited, so it may fall into the local optimum. By using the relaxation factor, p, the objective function becomes nonlinear with respect to $\lambda_{1}$ and $\lambda_{2}$, and each modality will give a contribution in the optimization process. The Lagrange function can be constructed as follows:

(13) $$L(\lambda ,\eta )=\lambda_{1}^{p}F_{1}+\lambda_{2}^{p}F_{2}+\beta F_{c}-\eta (\lambda_{1}^{}+\lambda_{2}^{}-1)$$

By setting $\frac{\partial L(\lambda ,\eta )}{\partial \lambda }$ and $\frac{\partial L(\lambda ,\eta )}{\partial \eta }$ to 0, $\lambda_{k}$ can be updated as:

(14) $$\lambda_{k}=\frac{{\left(1/F_{k}\right)}^{1/(p-1)}}{{\left(1/F_{1}\right)}^{1/(p-1)}+{\left(1/F_{2}\right)}^{1/(p-1)}},\;k=1,2$$

In our fusion network, the discriminative terms and the correlation term of the two modalities are back-propagated to the two CNNs. Given the optimized $\lambda_{k}$, the back-propagation can be conducted using the following derivatives of F with respect to $f_{1}(x_{i})$ and $f_{2}(x_{i})$:

(15) $$\frac{\partial F}{\partial \beta }=F_{c}$$

(16) $$\frac{\partial F}{\partial \mu }=\frac{\partial F_{c}}{\partial \mu }$$

(17) $$\frac{\partial F}{\partial f_{1}(x_{i})}=\lambda_{1}^{p}\frac{\partial F_{1}}{\partial f_{1}(x_{i})}+\beta \frac{\partial F_{c}}{\partial f_{1}(x_{i})}$$

(18) $$\frac{\partial F}{\partial f_{2}(x_{i})}=\lambda_{2}^{p}\frac{\partial F_{2}}{\partial f_{2}(x_{i})}+\beta \frac{\partial F_{c}}{\partial f_{2}(x_{i})}$$

The learning steps of the proposed multi-modal neural network can be listed as follows:

1. Initialize the RGB CNN and the depth CNN with parameters from the AlexNet, which has been pre-trained on the ImageNet large scale dataset.
2. Train the RGB CNN and the depth CNN, respectively, using the SGD method with back-propagation.
  For the RGB CNN,
  (1) Update $\rho_{1}$ according to Equation (2).
  (2) Update the parameters in the RGB CNN according to Equations (3)–(6).
  (3) Repeat (1)–(2) until convergence or the maximum number of iterations is reached.
  Likewise, for the depth CNN. The parameter, $\rho_{2}$, and the parameters in the RGB CNN are updated in turn.
3. Train the fusion network using the SGD method with back propagation.
  (1) Update $\lambda_{k}$ according to Equation (14).
  (2) Update β according to Equations (8) and (15).
  (3) Update μ according to Equations (9) and (16).
  (4) Update the parameters in the two CNNs according to Equations (17) and (18).
    Repeat (1)–(2) until convergence or the maximum number of iterations is reached.

### 3.3. RGB-D Object Recognition Based on DS Evidence Theory

**Figure 4:**
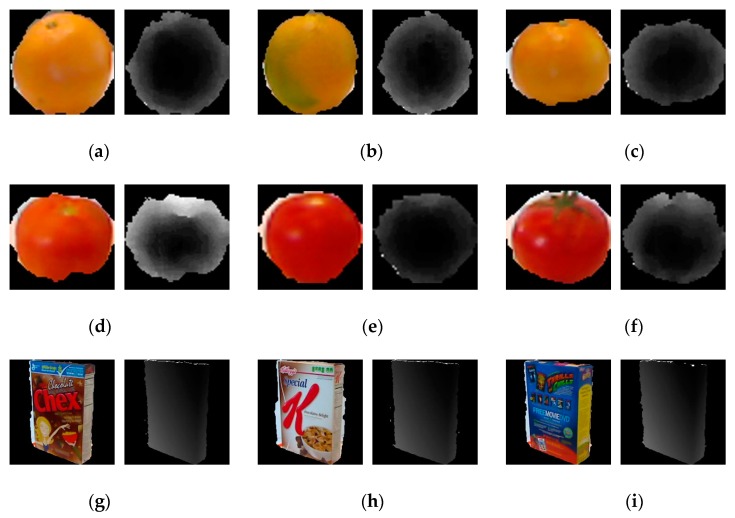

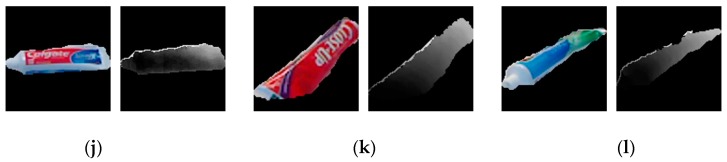
Examples of the RGB images and the depth images. (**a**–**c**) Three samples from the class “orange”; (**d**–**f**) three samples from the class “tomato”; (**g**–**i**) three samples from the class “cereal_box”; (**j**–**l**) three samples from the class “toothpaste”.

In this paper, we designed two SVM classifiers for the RGB modality and the depth modality, and used the DS evidence theory to fuse the two decision results. As shown in Figure 4, for different classes, the effective information of each modality has different proportions. Figure 4a–c are three samples from the class “orange”, and Figure 4d–f are three samples from the class “tomato”. We can see that the two classes of samples have different color information, but they have similar shapes. So their RGB images are discriminative and their depth images are similar. For the recognition task of the two classes, the RGB information is more important than the depth information. Figure 4g–i are three samples from the class “cereal_box”, and Figure 4j–l are three samples from the class “toothpaste”. We can see that for the same class, they have different color information and similar depth information. So, for the above two classes, the depth images have more effective discriminative information than the RGB images. Under this condition, the depth information is more important than the RGB information. From the above analysis, we can conclude that for different classes, the RGB modality and the depth modality have different contributions for recognition. It is not reasonable to weight the two SVM outputs directly. So, we used the DS evidence theory to fuse the decisions of the SVMs.

As the standard SVM algorithms cannot provide the posterior probability for post-processing, we adopted the sigmoid SVM to extract probabilities from SVM outputs [46]. The method can map the SVM outputs into probabilities by an additional sigmoid function. The class-conditional densities between the margins can be expressed using a parametric form of a sigmoid function:

(19) $$P(y=1|f)=\frac{1}{1+\exp(Af+B)}$$

where as long as A<0, the monotonicity of Equation (19) is assured. The parameters, A and B, can be found by minimizing the negative log likelihood of the training data, which is defined as:

(20) $$\min-\sum_{i}t_{i}\log(p_{i})+(1-t_{i})\log(1-p_{i})$$

where $p_{i}=\frac{1}{1+\exp(Af_{i}+B)}$, $t_{i}=\frac{y_{i}+1}{2}$. So, for each testing sample, we can obtain c probability outputs using the sigmoid SVM method, where c is the number of the classes.

For the RGB modality, we can obtain the probability outputs, $\left\{p_{1}^{\left(1\right)},p_{1}^{\left(2\right)},\cdots ,p_{1}^{\left(c\right)}\right\}$, of the testing sample, x, from the first classifier (SVM1). Then, the trust degree of the sample, x, can be defined as:

(21) $$m_{1}(x\in \omega_{i})=\alpha_{1}p_{1}^{\left(i\right)}+\alpha_{2}\frac{1}{c-1}\left[1-\frac{F_{1}-FM_{1}^{\left(i\right)}}{\sum_{j=1}^{c}\left(F_{1}-FM_{1}^{\left(j\right)}\right)}\right],\;i=1,2,\cdots ,c$$

where $\alpha_{1}$ and $\alpha_{2}$ are weights, which satisfy $\alpha_{1}+\alpha_{2}=1$. $p_{1}^{\left(i\right)}$ is the ith probability outputs of the SVM1, which means the probability of $x\in \omega_{i}$. To enhance the robustness and the effectiveness of the algorithm, we added the second item based on the RGB features. In Equation (21), $F_{1}$ is the RGB feature of the sample, x, and $FM_{1}^{i}$ is the mean value of the RGB features of the samples from class $\omega_{i}$. From Equation (21), we can conclude that the greater the value of $p_{1}^{\left(i\right)}$ is, the higher the trust degree becomes. Similarly, the second item is also proportional to the trust degree. So, for the testing sample, x, we can obtain c trust degrees of the RGB modality.

For the depth modality, the trust degree of the sample, x, can be defined using a similar method. It can be expressed as:

(22) $$m_{2}(x\in \omega_{i})=\alpha_{1}p_{2}^{\left(i\right)}+\alpha_{2}\frac{1}{c-1}\left[1-\frac{F_{2}-FM_{2}^{\left(i\right)}}{\sum_{j=1}^{c}\left(F_{2}-FM_{2}^{\left(j\right)}\right)}\right],\;i=1,2,\cdots ,c$$

where $p_{2}^{\left(i\right)}$ is the ith probability outputs of the SVM2, $F_{2}$ is the depth feature of the sample, x, and $FM_{2}^{i}$ is the mean value of the depth features of the samples from class $\omega_{i}$.

Then, the total trust degree of the sample, x, can be obtained based on the Dempster rule of combination. That is:

(23) $$m(x\in \omega_{k})=\left(m_{1}⊕m_{2}\right)(x\in \omega_{k})=\frac{\sum_{A\cap B=(x\in \omega_{k})}m_{1}(A)m_{1}(B)}{\sum_{A\cap B\neq \Phi }m_{1}(A)m_{1}(B)},\;i=1,2,\cdots ,c$$

Finally, for the testing sample, x, we compared its c total trust degrees and assign it to the class with the greatest total trust degree.

## 4. Experimental Results

### 4.1. Dataset and Implementation Details

**Figure 5:**
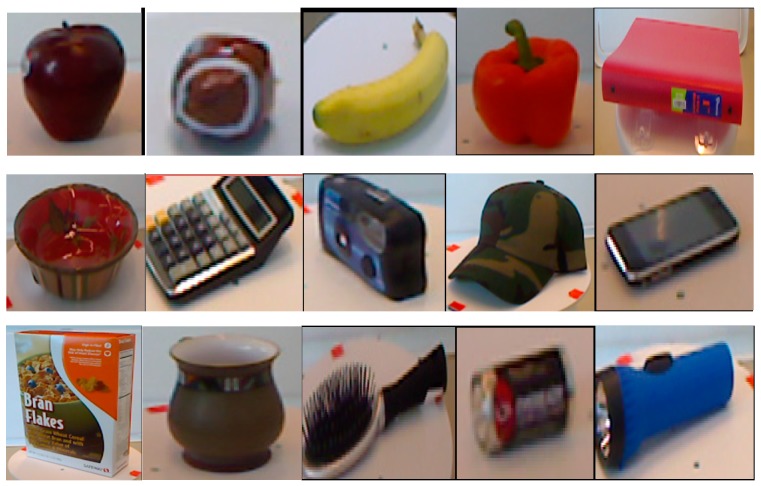
Objects of different categories from the Washington RGB-D object dataset.

In this paper, we used the Washington RGB-D object dataset to evaluate the proposed RGB-D object recognition method [21]. The dataset contains 300 household objects organized into 51 categories, which is collected using a sensing apparatus consisting of a prototype RGB-D camera manufactured by Prime-Sense and a firewire camera from Point Grey Research. Figure 5 shows some objects of different categories from the Washington RGB-D object dataset. Each object was captured with the cameras mounted at three different heights from three different directions. There are in total 207,920 RGB-D images, with about 600 images per object. The object recognition experiments in this paper are focused on category recognition. Following the experimental setting in Ref. [21], we subsampled the images at every 5th frame and used the same 10 cross-validation splits to evaluate the proposed method. Each split consists of roughly 35,000 training images and 7000 testing images. We averaged 10 recognition accuracies as the final results.

In the implementation, we first trained the proposed multi-modal network. The training stage had the following three steps: (1) Rescale the RGB images and the depth images to 227 × 227, and encode the depth images using the HHA encoding method; (2) train the RGB CNN and the depth CNN, respectively; (3) train the multi-modal network. Then, we validated the proposed recognition method using the testing samples. To obtain the recognition result of each testing sample, there were three steps: (1) Compute the RGB feature and the depth feature using the trained CNNs; (2) compute the trust degrees of the classification result of the two SVMs according to Equation (19) and Equation (20); (3) compute the total trust degree using Equation (21), and then we obtained the final recognition results. The RGB CNN and the depth CNN were initialized using the trained AlexNet on the ImageNet dataset. The weights of the CNNs were initialized using the pretrained network. The learning rate was first set to 0.01 and updated to 0.001 with the performance growth. The batch size, N, was set to 128. Table 1 gives 10 recognition accuracies of our proposed method using 10 cross-validation splits. From Table 1, we can see that the 10 recognition accuracies are similar to the mean value of them, and the variance of them is relatively small. So our proposed method is robust for different splits. In the following experimental results of this paper, we directly give the mean and standard deviation values of 10 recognition results.

### 4.2. Comparasion with Different Baselines

To validate the effectiveness of the multi-modal feature learning strategy and the DS evidence theory based decision fusion strategy proposed in this paper, we conducted experiments on the RGB images and the depth images with the following five different baselines:

1. RGB CNN: Used the CNN for learning RGB features and added a softmax layer to the end of the network for classification.
2. Depth CNN: Used the CNN for learning depth features and added a softmax layer to the end of the network for classification.
3. RGB CNN+SVM: Only trained the RGB CNN using the RGB images, and used the SVM as the classifier.
4. Depth CNN+SVM: Only trained the depth CNN using the depth images, and used the SVM as the classifier.
5. RGB-D CNNs+Multi-modal learning: The RGB CNN and the depth CNN are first trained using the RGB images and the depth CNN, respectively. Then, we performed multi-modal learning using Equation (12). Finally, the RGB feature and the depth feature are connected directly, and we sent the connected feature to the SVM classifier for object recognition.
6. RGB-D CNNs+DS fusion: The RGB CNN and the depth CNN were first trained, respectively. Then, the RGB feature and the depth feature were sent to the SVMs, respectively. Finally, our DS fusion strategy was used to fuse the two recognition results.
7. RGB-D CNNs+Multi-modal learning+DS fusion: Our proposed method.

**Figure 6:**
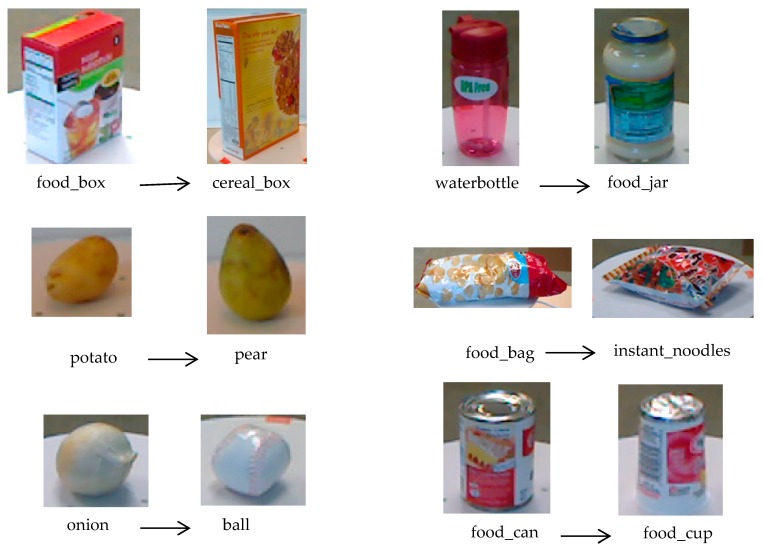
Examples of misclassified samples of the proposed method.

Table 2 shows the recognition accuracy of the five baselines on the Washington RGB-D object dataset, and we have made the score of the best method bold. From Table 2, we can see that compared with the “RGB CNN” method and the “Depth CNN” method, the “RGB+SVM” method and the “Depth+SVM” method performs better. So, we can conclude that the SVM classifier is better than the CNN based classifier. These results are similar to the RCNN based object detection method proposed in Ref. [47]. So, we adopted the SVM as the classifier in our proposed method. Compared with single modality based methods, the latter three RGB-D image based methods perform significantly better. This is because the identification information contained in the RGB image and the depth image has certain complementarity. Both the multi-modal learning strategy and the DS evidence theory based decision fusion strategy can improve the recognition performance. The recognition accuracy of the “RGB-D CNNs+DS fusion” method is lower than the “RGB-D CNNs+Multi-modal learning” method. So, we can conclude that our multi-modal learning strategy is more effective than our proposed DS evidence theory based decision fusion strategy. We can extract more effective discriminative features from the RGB-D images using our proposed multi-modal learning method. The “RGB-D CNNs+Multi-modal learning+DS fusion” method has the best performance. However, there are some classes, which are often misclassified. Figure 6 shows some misclassified samples of our proposed method. From Figure 6, we can see that the misclassifications are mainly due to a similar color and shape among samples from different classes. So, using the multi-modal learning strategy and the DS evidence theory based decision fusion strategy simultaneously can greatly improve the recognition accuracy.

### 4.3. Comparasion with State-of-the-Art Methods

We compared the recognition accuracy of our proposed method with the following 11 state-of-the-art methods: (1) Linear SVM [21]: Spin images and SIFT descriptors are used for depth feature extraction, and texton histograms and color histograms are used for RGB feature extraction. The linear support vector machine is used for classification. (2) Nonlinear SVM [21]: The adopted features are the same as those used in the “Linear SVM” method, and the Gaussian kernel SVM is used for classification. (3) HKDES [48]: The combination of hierarchical kernel descriptors are used for extracting the RGB and depth features, and the linear SVM is used for classification. (4) Kernel Descriptor [22]: A set of kernel descriptors are used for feature extraction, and linear SVM is used for classification. (5) CNN-RNN [30]: A model based on a combination of CNN and RNN is used for learning features and classifying RGB-D images. (6) RGB-D HMP [25]: The HMP method is used to learn hierarchical feature representations from raw RGB-D data in an unsupervised way. (7) MMSS [49]: A CNN based multi-modal sharable and specific feature learning framework is used for RGB-D object recognition. (8) Fus-CNN (HHA) [50]: A two-stream convolutional neural network is used for RGB-D feature extraction, and the HHA encoding method is used for depth images. (9) Fus-CNN (jet) [50]: A network the same as the “Fus-CNN (HHA)” method is used for feature extraction, but the jet encoding method is used for depth images. (10) CFK [51]: A convolutional Fisher Kernels (CFK) method is used for recognition, which integrates the advantages of CNN and Fisher Kernel encoding (FK). (11) MDCNN [32]: A three-stream multi-modal CNNs based deep network is used for RGB-D object recognition, and both the surface normal and jet color are used to encode the depth images. (12) VGGNet+3D CNN+ VGG3D [34]: The outputs for the pretrained VGGNet, the 3D CNN, and VGG3D architectures are fused, and the concatenated feature are sent to the linear SVM for final RGB-D object recognition.

The comparison results are illustrated in Table 3, and we have made the score of the best method bold. It can be seen that compared with the state-of-the-art RGB-D object recognition methods, our proposed method achieved competitive results. For RGB image based object recognition, the “VGGNet+3D CNN+ VGG3D” method achieved the best recognition accuracy, which uses the 16-layer VGGNet to learn features from RGB images. The scale of the deep network is larger than that of the CNN used in our proposed method, and its recognition result is slightly better than our proposed method. For the depth image based object recognition, the “CFK” method performed best. It first uses a CNN to learn translation ally invariant depth features, and then 3D spatial pyramids are applied to further improve the Fisher vector representation of depth modality. Compared with our proposed method, it can use effective spatial information for recognition. For RGB-D object recognition, our proposed method was generally better than most of the state-of-the-art methods. The performance of our proposed method is almost the same as that of the “VGGNet+3D CNN+ VGG3D” method, and it is slightly surpassed by the “MDCNN” method. The “MDCNN” method use a three-stream network, where RGB images are fed into one stream and depth images are fed into the other two streams. It uses a joint depth encoding technique, and the depth images are encoded into two categories: Jet color and 3D surface normal. Compared with the “MDCNN” method, our proposed method only uses one kind of depth encoding method. Furthermore, our proposed multi-modal network only has two streams, whose size is smaller than that of the “MDCNN” method. So, our proposed network is more efficient and more easily converges. Compared with the state-of-the-art methods, our proposed method has two information fusion steps. First, we performed multi-modal learning to learn both the discriminative features and the correlation features of the RGB and depth modalities. Second, we designed the DS evidence theory based decision fusion scheme to effectively integrate the classification results of the two SVMs. So, our proposed method can extract the effective information form the RGB-D images by multi-modal feature learning, and it achieved a better recognition performance without increasing the network size.

## 5. Conclusions

In this paper, we presented a novel RGB-D object recognition method based on multi-modal feature learning and the DS evidence theory. First, the RGB images and the depth images were preprocessed, and the HHA encoding method was used for obtaining three inputs for the following CNN. Next, two CNNs were, respectively, trained for initialization. Then, the multi-modal feature leaning was performed using our proposed objective function. Our proposed objective function uses the quadruplet samples to minimize intro-class distances and maximize inter-class distances, which has better performance and robustness. Furthermore, both the discriminative information of each modality and the correlation information between the two modalities were considered in our proposed objective function. Finally, two sigmoid SVMs were used to obtain probabilities, and the DS evidence theory was used for fuse the two classification results. Compared with other RGB-D object recognition method, our proposed method uses two fusion strategies to make full use of the effective information of the two modalities. Our extensive experimental results have shown that both the multi-modal feature learning and the DS evidence theory based decision fusion can effectively improve the performance of RGB-D object recognition. Compared with state-of-the-art methods, our proposed method achieved competitive results.

## Figures and Tables

**Table 1:** 10 recognition accuracies of our proposed method.

1	2	3	4	5	6	7	8	9	10	Mean	Var
92.9	92.7	90.1	91.9	92.2	90.4	93.1	90.2	91.7	92.8	91.8	1.4

**Table 2:** Comparison of different baselines on the Washington RGB-D object dataset.

Method	Accuracy (%)
RGB CNN	85.7 ± 2.3
Depth CNN	81.3 ± 2.2
RGB CNN+SVM	87.5 ± 2.1
Depth CNN+SVM	84.8 ± 2.0
RGB-D CNNs+Multi-modal learning	90.2 ± 1.8
RGB-D CNNs+ DS fusion	88.9 ± 1.9
RGB-D CNNs+Multi-modal learning+DS fusion	**91.8 ± 1.4**

**Table 3:** Comparison with state-of-the-art methods on the Washington RGB-D object dataset.

Method	Accuracy (%)		
	RGB	Depth	RGB-D
Linear SVM [21]	74.3 ± 3.3	53.1 ± 1.7	81.9 ± 2.8
kSVM [21]	74.5 ± 3.1	64.7 ± 2.2	83.8 ± 3.5
HKDES [48]	76.1 ± 2.2	75.7 ± 2.6	84.1 ± 2.2
Kernel Descriptor [22]	77.7 ± 1.9	78.8 ± 2.7	86.2 ± 2.1
CNN-RNN [30]	80.8 ± 4.2	78.9 ± 3.8	86.8 ± 3.3
RGB-D HMP [25]	82.4 ± 3.1	81.2 ± 2.3	87.5 ± 2.9
MMSS [49]	74.6 ± 2.9	75.6 ± 2.7	88.5 ± 2.2
Fus-CNN (HHA) [50]	84.1 ± 2.7	83.0 ± 2.7	91.0 ± 1.9
Fus-CNN (Jet) [50]	84.1 ± 2.7	83.8 ± 2.7	91.3 ± 1.4
CFK [51]	86.8 ± 2.2	**85.8 ± 2.3**	91.2 ± 1.5
MDCNN [32]	87.9 ± 2.0	85.2 ± 2.1	**92.2 ± 1.3**
VGGnet + 3D CNN + VGG3D [34]	**88.9 ± 2.1**	78.4 ± 2.4	91.8 ± 0.9
Our proposed method	87.5 ± 2.1	84.8 ± 2.0	91.8 ± 1.4
